# The Role of Mitochondrial NADPH-Dependent Isocitrate Dehydrogenase in Cancer Cells

**DOI:** 10.1155/2012/273947

**Published:** 2012-05-20

**Authors:** Katarína Smolková, Petr Ježek

**Affiliations:** Department of Membrane Transport Biophysics (No. 75), Institute of Physiology v.v.i., Academy of Sciences of the Czech Republic, Vídeňská 1083, CZ-14220 Prague, Czech Republic

## Abstract

Isocitrate dehydrogenase 2 (IDH2) is located in the mitochondrial matrix. IDH2 acts in the forward Krebs cycle as an NADP^+^-consuming enzyme, providing NADPH for maintenance of the reduced glutathione and peroxiredoxin systems and for self-maintenance by reactivation of cystine-inactivated IDH2 by glutaredoxin 2. In highly respiring cells, the resulting NAD^+^ accumulation then induces sirtuin-3-mediated activating IDH2 deacetylation, thus increasing its protective function. Reductive carboxylation of 2-oxoglutarate by IDH2 (in the reverse Krebs cycle direction), which consumes NADPH, may follow glutaminolysis of glutamine to 2-oxoglutarate in cancer cells. When the reverse aconitase reaction and citrate efflux are added, this overall “anoxic” glutaminolysis mode may help highly malignant tumors survive aglycemia during hypoxia. Intermittent glycolysis would hypothetically be required to provide ATP. When oxidative phosphorylation is dormant, this mode causes substantial oxidative stress. Arg172 mutants of human IDH2—frequently found with similar mutants of cytosolic IDH1 in grade 2 and 3 gliomas, secondary glioblastomas, and acute myeloid leukemia—catalyze reductive carboxylation of 2-oxoglutarate and reduction to D-2-hydroxyglutarate, which strengthens the neoplastic phenotype by competitive inhibition of histone demethylation and 5-methylcytosine hydroxylation, leading to genome-wide histone and DNA methylation alternations. D-2-hydroxyglutarate also interferes with proline hydroxylation and thus may stabilize hypoxia-induced factor *α*.

## 1. Oxidative Phosphorylation and Glutaminolysis in Cancer Cells

### 1.1. Strategies for Survival of Malignant Tumors

During malignant transformation, cells undergo stages of gene expression reprogramming and mutagenesis that alter their metabolic phenotype(s) [1–5]. Initial stimuli (not all known) dysregulate information signaling and activate oncogenes and/or cancer stem cells, resulting in a partial glycolytic “Warburg” phenotype [1–5] in which pyruvate is diverted, at least to a certain extent, from oxidative phosphorylation (OXPHOS). High proliferation and impaired angiogenesis subsequently cause hypoxia in certain regions within a growing tumor, and then hypoxia-mediated metabolic reprogramming (such as that promoted by hypoxia-induced factor, HIF [6–8]) further intensifies the glycolytic phenotype and may nearly completely divert pyruvate from pyruvate dehydrogenase (PDH), that is, from OXPHOS. The sustained high rate of cell proliferation, however, results in aglycemia, initiating the revival of OXPHOS in conjunction with the promotion of glutaminolysis [1, 2, 9, 10]. The overall glutaminolysis provides cytosolic pyruvate/lactate and also yields NADPH via citrate export from mitochondria and subsequent ATP-citrate lyase and malic enzyme reactions. This compensates for the reduced net energy production by the glycolytic pathway and pentose phosphate pathway (PPP). Pyruvate imported into mitochondria is the precursor of not only acetyl-CoA but also citrate, which is required for fatty acid synthesis and hence for phospholipid synthesis, so it is essential for cell growth [1–5]. The final established phenotype is exemplified by human glioblastoma cells, which, despite their low respiration, maintain a constant pyruvate flux through PDH and hence partial OXPHOS [9]. Oxaloacetate, however, may also be provided by the pyruvate carboxylase reaction [11].

### 1.2. Glutaminolysis at Rejuvenated OXPHOS

For the purpose of this paper, we shall use the term “glutaminolysis” in a more general way than just the transformation of glutamine to 2-oxoglutarate (2OG). We categorize glutaminolysis according the fate of 2OG after its initial formation from glutamine [1]. If 2OG resulting from glutamine acts in the forward Krebs cycle (despite possible ongoing citrate extrusion and truncation of the cycle so that aconitase and “classic” NAD^+^-dependent isocitrate dehydrogenase (IDH3) reactions are not required), we define the system of metabolic reactions involved as “OXPHOS glutaminolysis.” This term points out to its essential dependence on succinate dehydrogenase (Complex II) and hence on respiration and OXPHOS. In contrast, when the reductive carboxylation of 2OG by isocitrate dehydrogenase 2 (IDH2) (in the counter Krebs cycle direction) consuming NADPH follows glutaminolysis of glutamine to 2OG and when the reverse aconitase reaction and citrate efflux are added, we define that system as “reductive carboxylation glutaminolysis” (RCG), also referred to as “anoxic glutaminolysis.” The latter term denotes the absolute independence of oxygen (respiration).

 In general, glutaminolysis is an anaplerotic pathway of the Krebs cycle. Although it acts frequently in broad cancer types, glutaminolysis is not universal for all cancers [3–5]. In cancer cells employing OXPHOS glutaminolysis, glutamine can fully compensate for the lack of glucose in terms of energy generation and syntheses of precursors for anabolic pathways [3–5]. Thus, to survive under conditions of limited glucose, highly glycolytic cancer cells may adapt to glutaminolysis, which in its OXPHOS mode restores OXPHOS and may restore also at least partial PDH function [1, 3, 12–14]. In normal cells, mitochondrial glutaminase catabolizes glutamine to produce ammonia and glutamate, which is further transaminated by glutamate dehydrogenase into 2OG to feed the Krebs cycle [15]. In malignant tumors, negative allosteric effectors, such as GTP, inhibit glutamate dehydrogenase, resulting in a move toward glutaminolysis, where glutamate and pyruvate are reactants in a transamination reaction that produces, for example, alanine and 2OG by alanine aminotransferase (transaminase) [15]. In cancer cells, 2OG usually feeds the forward-running Krebs cycle truncated after citrate synthase during citrate extrusion, so that aconitase and “classic” NAD^+^-dependent IDH3 reactions are not required [1, 5]. This OXPHOS glutaminolytic mode is strictly dependent on oxaloacetate and acetyl-CoA, that is, on the citrate synthase reaction; hence, it proceeds only in cells in which oxaloacetate is provided by malate dehydrogenase fed by the Krebs cycle as well as by malate import from the cytosol, where malate originates from ATP-citrate lyase reaction. Likewise incomplete inhibition of PDH restores acetyl-CoA in mitochondria (the pyruvate pool is split between the PDH and transaminase reactions). An alternative oxaloacetate source is provided by pyruvate carboxylase reaction [11].

Also, the mitochondrial malic enzyme may contribute to this pool by producing pyruvate from malate [16]. Citrate is extruded from mitochondria and converted to oxaloacetate and acetyl-CoA by ATP citrate lyase [17]. Acetyl-CoA is then used to produce fatty acids by fatty acid synthase and cholesterol for general lipid synthesis, which is essential for cancer cell proliferation [18, 19]. In glioblastoma, if there is excess NADH in the cytoplasm (produced by aerobic glycolysis), the cytosolic oxaloacetate is converted first to malate by malate dehydrogenase and then to pyruvate by the cytosolic malic enzyme, thus may also contribute to lactate production [9, 20]. The cytosolic malic enzyme also produces NADPH as another factor required for lipid biosynthesis. Moreover, alanine that is released by transaminase is used for cytosolic amino acid transformations and protein synthesis [1, 4, 5].

### 1.3. Glutaminolysis Independent of OXPHOS

Hypothetically, malignant tumors may survive on intermittent OXPHOS-independent RCG (also termed “anoxic” glutaminolysis) in parallel with intermittent glycolytic periods [1, 2]. RCG utilizes reductive carboxylation of 2OG by the reverse reaction of mitochondrial IDH2 at the expense of NADPH, followed by the reverse aconitase reaction and citrate efflux from the matrix [1–3, 21–23]. NADPH is provided by the malic enzyme converting malate to pyruvate and might also be provided by the mitochondrial transhydrogenase [24]. The OXPHOS independence of this mode means that it may proceed at any level of hypoxia and even at anoxia, thus increasing malignancy [1, 3]. However, it does not produce ATP so parallel glycolytic periods are required [1]. The reductive carboxylation involves IDH2, which converts 2OG to isocitrate, from which the reverse aconitase reaction produces citrate, which is again exported from the mitochondrial matrix to the cytosol for fatty acid and lipid synthesis. Note that acetyl-CoA, and hence the PDH reaction, is not required in this mode.

## 2. Isocitrate Dehydrogenase Enzyme Isoforms

### 2.1. Overview of IDH Isoforms

All eukaryot genomes contain three *IDH* genes. *IDH3* encodes a mitochondrial matrix NAD^+^-dependent octameric IDH3 (4*α*2*β*2*γ* subunits [25]) that acts in the Krebs cycle. IDH3 is allosterically positively regulated by Ca^2+^, ADP, and citrate and negatively regulated by ATP, NADH, and NADPH. The two other *IDH* genes, *IDH1 *and *IDH2*, encode cytosolic and mitochondrial matrix NADP^+^-dependent (or NADPH-dependent) IDH1 and IDH2, respectively, which are structurally and genetically unrelated to IDH3 [26] (Table 1). IDH3 irreversibly decarboxylates isocitrate to yield 2OG while reducing NAD^+^ to NADH, whereas IDH1 and IDH2 catalyze reversible reactions, either decarboxylating isocitrate to 2OG while reducing NADP^+^ to NADPH or acting in the reductive carboxylation reaction to convert 2OG to isocitrate while oxidizing NADPH to NADP^+^.

Heterozygous mutations in *IDH2* at Arg172 and at the analogous residue Arg132 in *IDH1* are frequently found in grade 2 and 3 gliomas, secondary glioblastomas, and acute myeloid leukemia (AML [27]), but they occur less frequently in primary glioblastomas and other cancers [28–37]. No homozygous deletions of *IDH1 *and *IDH2* have been found, as has been observed for classic tumor suppressors. Nevertheless, mutated IDH1 and IDH2 exhibit a neomorphic enzyme activity, reducing 2OG to d-2-hydroxyglutarate while converting NADPH to NADP^+^[23, 29, 36, 38–40]. Interestingly, the d-2-hydroxyglutarate thus formed further promotes neoplasia by competitive inhibition of histone demethylation and 5-methyl-cytosine hydroxylation, leading to genome-wide alternations in the methylation of histones and DNA [40]. It has also been reported that glioblastoma SF188 cells produce d-2-hydroxyglutarate, in spite of lacking the above-described mutations [41].

Moreover, IDH2, like ~20% of other mitochondrial enzymes [42, 43], is acetylated at lysines, which inactivates the enzymatic activity. In turn, deacetylation of IDH2 by the mitochondrial matrix deacetylase sirtuin 3 (SIRT3) activates the enzyme to produce more NADPH [44]. In nonmalignant cells, the cytosolic IDH1 is involved in lipid metabolism and glucose sensing. IDH2 was traditionally considered to be involved in the regulation of OXPHOS and redox homeostasis [45] (see Section 4), and its involvement in reductive carboxylation has been recognized only recently (Sections 2.4 and 3).

### 2.2. Specific Enzymatic Properties of IDH2

**Figure 1:**
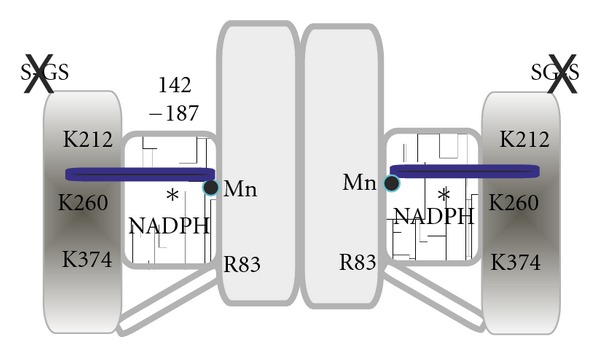
Main domains of the mitochondrial isocitrate dehydrogenase, IDH2.The homodimeric IDH2 of two 413-amino acid subunits; each 47 kDa subunit is schematically illustrated according the solved crystal structure of porcine IDH2 [50]. Residues 142–187 make up two stacked four-stranded antiparallel *β*-sheets (middle white marble-shaded domain). Mn^2+^ and isocitrate in the active site are depicted as a blue circle and rod, respectively. Six Arg residues, which provide hydrogen bonds with isocitrate oxygens [50], are not depicted. Arg 83 (R83) interacts by hydrogen bonding with the 3′-OH of the nicotinamide ribose and thus enhances NADP^+^ affinity [53]. An Arg residue corresponding to human Arg172, which is often mutated in gliomas and AML, is indicated by an asterisk (∗). For activity, the glutathionylation of Cys269 [46] (GS–S) must be removed (X) by reactivation aided by glutaredoxin-2 in the presence of reduced glutathionine, and the protein must be deacetylated by SIRT3 [44]. We hypothesize that Lys212, Lys374, and Lys260 (in porcine notation) are the prime candidates for acetylation causing IDH2 inactivation.

NADP^+^-dependent oxidative decarboxylation of isocitrate to 2OG, as the major function of IDH2 in nonmalignant cells, contributes substantially to the control of mitochondrial redox balance and the prevention of oxidative damage [45, 46]. Because the IDH2 reaction is reversible, it may act in a “reverse” Krebs cycle mode in the reductive carboxylation reaction (see Section 2.4). IDH2 contains an N-terminal mitochondrial addressing sequence and hence is imported to the mitochondrial matrix [45], although localization to nuclei has also been reported [47]. The *IDH2* locus is adjacent to the gene for the *α* subunit of IDH3 [48]. IDH2 expression in heart, skeletal muscle, and lymphocytes is quite substantial; lower levels are found in liver, kidney, and lung [45, 47]. IDH2 has also been found in cultured rat neurons, astrocytes, oligodendrocytes, and microglia [49]. Unlike IDH1, the 94-kDa IDH2 (EC 1.1.1.42) is a homodimeric enzyme of two 413-amino acid subunits, each 47 kDa [50, 51] (Figure 1). IDH2 function requires a divalent metal ion, and bound Mn^2+^ yields the maximum activity [52]. The structure of the Mn^2+^-isocitrate binding site was mapped from the solved crystal structure of porcine IDH2 [50]. Within the site, Thr78, Ser95, and Asn97 (of the porcine sequence) donate a hydrogen bond to the C3 carboxyl, whereas Asp252 and Asp275 coordinate Mn^2+^. An additional six Arg residues provide hydrogen bonds with isocitrate oxygens [50]. Hydrogen bonding of Lys212 with other residues between the two subunits was also noted. The NADP^+^ binding site was originally predicted from the *E. coli* IDH structures, positioning the 2-hydroxyl-bound phosphate to interact with His315 and Lys374 of porcine IDH2 [50]. Porcine Arg83 enhances NADP^+^ affinity by hydrogen bonding with the 3′-OH of the nicotinamide ribose, and Asn328 provides a hydrogen bond to the N1 of adenine [53]. For efficient coenzyme site function, a hydroxyl group must be present at position 373 (Thr373 of the porcine sequence), whereas Asp375 and Lys260 contribute to coenzyme affinity and catalysis [54, 55].

 Within the numbering of the human IDH2 sequence, mutations in Arg172 (an analog of frequently mutated Arg132 of cytosolic IDH1 [56]) are detected in gliomas [23, 36, 37], and mutations in Arg172 and Arg140 (which is adjacent in the active site to Arg172 [50]) are found in AML [57]. Mutations are apparent after the transition from a normal cell to a clinically evident tumor. Arg172 (as well as Arg132 of IDH1) provides hydrogen bonds to the *α* and *β* carboxyls of isocitrate and may be important in the transition from an open to closed state of the active site [50]. The porcine Arg residue mutated to Asn in a position analogous to human Arg172 displays a twofold reduction in specific activity and a K_*m*_ for isocitrate that is two orders of magnitude higher [51] (Table 1). Likewise, in lysates of cells overexpressing IDH2, activity is reduced when Arg172 is substituted with Gly, Lys, or Met [37]. Nevertheless, the existence of exclusively heterozygous IDH2 (and IDH1) mutations in gliomas and AML and the small possibility of dominant-negative mutations (minor mutant fractions existing could not exert this role [39]) have led to the search for other consequences of IDH1 and IDH2 mutations.

Arg132 mutants of cytosolic IDH1 [27] (Table 1) and Arg172 mutants of mitochondrial IDH2 [23, 36, 38, 39] possess the ability to reduce 2OG to d-2-hydroxyglutarate while converting NADPH to NADP^+^. This is because the active closed state of the enzyme exhibits a higher affinity for NADPH. Mutant IDH2 and IDH1 [27] are not supposed to allow the reductive carboxylation reaction of 2OG to isocitrate. However, consequences of these mutations have appeared more detrimental than expected. Initially, the production of d-2-hydroxyglutarate in gliomas, secondary glioblastomas, and AML by mutant IDH2 results in a decrease of 2OG and hence depletion of succinate, fumarate, and malate from the rest of the Krebs cycle [23, 36, 39]. Interestingly, IDH2 mutations also lead to increases in amino acid levels [35] as would be expected for ongoing glutaminolysis. However, the most profound consequences stem from the interference with epigenomics. Thus, the formed d-2-hydroxyglutarate strengthens the neoplastic phenotype by competitive inhibition of histone demethylation and 5-methyl-cytosine hydroxylation, leading to genome-wide alternations in histone and DNA methylation [40]. Moreover, prolyl hydroxylase domain enzymes, which employ 2OG as a cofactor for marking hypoxia-induced factor-1*α* (HIF1*α*) by proline hydroxylation, are inhibited by d-2-hydroxyglutarate as well as by the lack of 2OG. As a result, HIF1*α* is stabilized (if its inhibitor, aspartyl hydroxylase factor inhibiting HIF, is not active) even at normoxia and thus can elicit the otherwise hypoxic reprogramming of gene expression [58].

### 2.3. Regulation of IDH2 Activity

Glutathionylation of proteins by the reversible glutaredoxin (thioltransferase) reaction serves to protect against irreversible oxidation of cysteines [59]. Such protection has been demonstrated for IDH2 in that oxidized glutathione can inactivate IDH2 by forming a mixed disulfide bond with Cys269 [46]. The inactivated IDH2 is reactivated by glutaredoxin 2 in the presence of reduced glutathione. Also, IDH2 in mouse heart may exist in complex with calcineurin, containing as well aconitase, malate dehydrogenase, and MnSOD [55].

Another important level of regulation of IDH2 activity is provided by mitochondrial SIRT3. Posttranslational modifications of numerous mitochondrial proteins frequently occur via acetylation/deacetylation of lysine residues [42, 43, 60, 61]. Among seven sirtuin family members, SIRT3, SIRT4, and SIRT5 are enriched in mitochondria, such as exemplified and well described for SIRT3 up to date [42, 43, 62]. It has been reported that incubation of SIRT3 with IDH2 increases the dehydrogenase activity [62]. Recently, caloric restriction was demonstrated to act via IDH2 deacetylation through SIRT3 [44]. Caloric restriction prevents age-related hearing loss by reducing oxidative DNA damage, but such is not the case for mice lacking SIRT3 [44]. SIRT3 directly deacetylates the IDH2 lysines and thus activates IDH2, which in its forward mode could result in increased NADPH levels and thereby maintain reduced glutathione levels in mitochondria. Indeed, overexpression of SIRT3 and/or IDH2 leads to increased NADPH levels and is protective against oxidative stress-induced cell death [44]. Lys212, Lys374, and Lys260 (porcine sequence) may be the prime candidates for acetylation/inactivation. It remains to be shown that SIRT3 can deacetylate these residues, however, and it should be investigated which reaction modes are active and possible before and after such activation, specifically, whether the reverse, NADPH-dependent, reaction and RCG could be activated.

### 2.4. Reductive Carboxylation Reaction

As predicted in 1994 by Sazanov and Jackson [63] (see also [64]), the reductive carboxylation reaction by native IDH2 converts 2OG to isocitrate while oxidizing NADPH to NADP^+^. The Arg172 and Arg140 mutants of IDH2 [23, 36, 38, 39] and glioblastoma SF188 cells under hypoxia [41] convert 2OG to d-2-hydroxy-glutarate in this “reverse-reaction” mode. This reductive carboxylation would proceed better *in vivo* when followed by the reverse aconitase reaction and subsequent citrate export from the mitochondrial matrix. Reductive carboxylation was demonstrated for IDH2 in 2002 [65] and was indicated for cancer cells in transformed brown adipocytes [22], pediatric glioma SF188 cells [23, 41], and UOK262 cells (derived from a renal tumor in a patient with hereditary leiomyomatosis, these cells are defective in respiration and devoid of fumarate hydratase activity) [2]. Reductive carboxylation accompanied by citrate efflux has also been found in quiescent fibroblasts and is enhanced in contact-inhibited fibroblasts [66]. IDH2 silencing in SF188 cells results in diminished conversion of glutamine to citrate [23, 41]. Recently, reductive carboxylation was detected in human osteosarcoma 143B cells in which the mitochondrial DNA encoded a loss-of-function mutation in respiratory chain Complex III (CYTB 143B cells) [2]. Because only low-level reductive carboxylation was detected in wild-type 143B cells, the authors suggested that the impairment of OXPHOS, such as given by mutant mitochondrial DNA, induces RCG. Silencing of either IDH1 or IDH2 reduces the growth of both wild-type and CYTB 143B cells [2]. Moreover, unlike in wild-type 143B cells, *de novo* fatty acid synthesis from glutamine as a precursor is prevalent in CYTB 143B cells [2]. Reductive carboxylation in fumarate hydratase—devoid UOK262 cells, which are defective in respiration, has also been identified in parallel with OXPHOS glutaminolysis [2]. Interestingly, inhibition of respiration in mouse embryonic fibroblasts via administration of antimycin, rotenone, or metformin induces a switch towards RCG [2]. Thus, these data provide additional support for the authors' hypothesis that RCG is a common cellular response to impaired mitochondrial metabolism [2].

## 3. Contribution of IDH2 to Glutaminolysis That Is Independent of OXPHOS

### 3.1. Evidence for Reverse Reactions in the Krebs Cycle

The reverse IDH2 reaction was also considered such that IDH2 acts together with the forward reaction of IDH3 in a dissipative isocitrate/2OG cycle [63] (see Section 4.2). The reductive carboxylation reaction and the overall RCG may indeed proceed together with the forward decarboxylation reaction [2, 22]. The best evidence was obtained by tracking the metabolites of ^13^C-labeled glutamine, such as the appearance of ^13^C-label in citrate [2, 22, 23, 41].

### 3.2. RCG in Cancer Cells

The first demonstrations of RCG in cancer cells [22, 23, 41] are consistent with the recent findings that mutant IDH2 in gliomas and AML also produce d-2-hydroxyglutarate from 2OG by “alternate” reduction. Because these mutants are heterozygous, both RCG and the production of d-2-hydroxyglutarate might occur. The former reaction involves the nonmutant NADPH-dependent IDH2 “reverse” reaction followed by isocitrate conversion to citrate and by citrate export. The mutant IDH2 (but maybe also wild-type IDH2, see [41]) acting in a “reverse” mode also produces d-2-hydroxyglutarate, which cannot be transformed by aconitase; however, it further enhances the malignant phenotype [40]. The importance of this neomorphic IDH2 activity for the cancer phenotype is valid even without consideration of d-2-hydroxyglutarate interference with epigenetics and the HIF pathway, because IDH2 depletes 2OG from the Krebs cycle.

**Figure 2:**
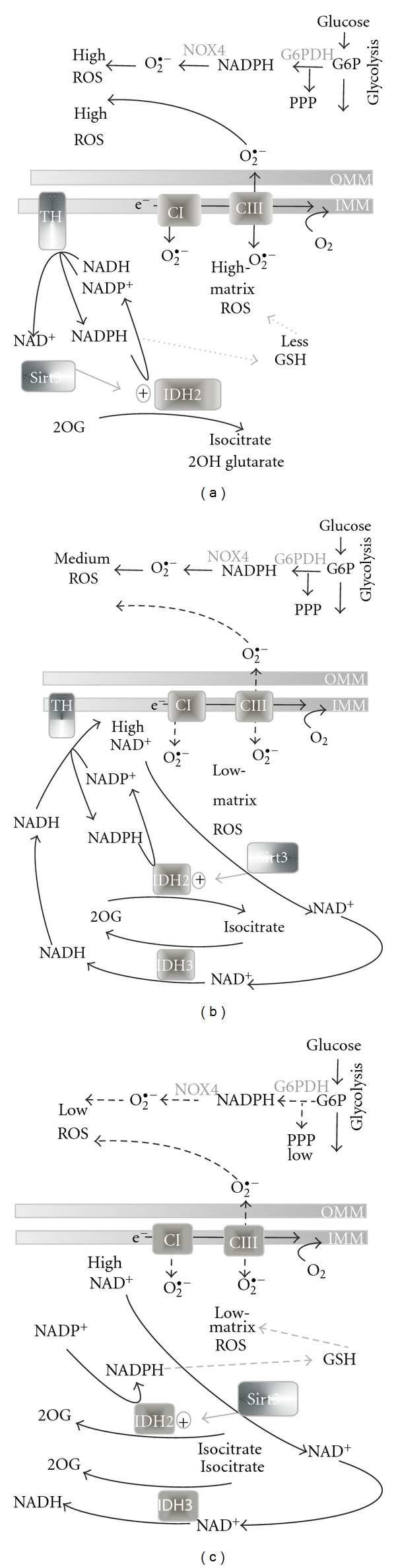
Consequences of IDH2 functions for redox homeostasis in cancer cells and nonmalignant cells.

 The consumption of NADPH in the matrix is a consequence of the altered homeostasis of reactive oxygen species (ROS) in cancer cells (see Section 4.2) and is also possible due to NADPH production by the mitochondrial malic enzyme [1, 5] and transhydrogenase [24, 63, 67]. It is not known whether SIRT3-based activation also affects this reverse (NADPH-dependent) IDH2 reaction. Although NADH, rather than NAD^+^, accumulates in the mitochondrial matrix of highly glycolytic cancer cells in which OXPHOS is dormant (Figure 2(a)), NAD^+^ might be produced by the inner membrane H^+^ transhydrogenase from NADH with the simultaneous formation of NADPH from NADP^+^ in the matrix [67], thereby activating SIRT3-mediated IDH2 deacetylation. Moreover, the usual acetylation of proteins may be retarded in highly glycolytic cancer cells; hence, no SIRT3-mediated deacetylation would be required.

### 3.3. Intermittent Nature of RCG

Unlike glycolysis, RCG does not form ATP. Hence, either RCG coexistence with glycolysis or intermittent glycolysis is expected under hypoxic and deep hypoxic conditions [1]; that is, RCG may help cancer cells survive aglycemia and hypoxia in malignant cells [68]. Nevertheless, before all ATP stores become consumed, glycolysis must be reestablished. If this happens, it may help the tumor cell survive even in anoxia. As clearly demonstrated by the examples of gliomas and AML with the oncogenic metabolite d-2-hydroxyglutarate, the establishment of RCG, even concomitantly with OXPHOS glutaminolysis (note that this mode does not require IDH2 and aconitase reactions), helps to accelerate the malignant phenotype. Recently, it has been demonstrated that hypoxia elevates RCG in SF188 cells in a HIF-dependent manner [41]. SF188 cells were able to proliferate at 0.5% O_2_ even if such hypoxic conditions substantially diminished glucose-dependent production of citrate, that is, OXPHOS and forward Krebs cycle participation [41].

## 4. Role of IDH2 in ROS Homeostasis

### 4.1. Regulation of ROS Homeostasis in Nonmalignant Cells

A major function of IDH2 in nonmalignant cells, when acting within the forward Krebs cycle, is likely maintaining an adequate pool of reduced glutathione and peroxiredoxin by providing NADPH. This function improves the mitochondrial redox balance and prevents oxidative damage [45, 46, 69], including heat-shock-induced oxidative damage [70] and numerous consequent events of oxidative stress, such as ROS-induced apoptosis [71, 72], apoptosis induced by ionizing radiation [73] and cadmium [74], and staurosporine-induced cell death [75]. The lack of IDH2 or its activity elevates cytosolic ROS, lipid peroxidation, and oxidative DNA damage and shortens cell survival after oxidant exposure [69, 71–73]. Also, susceptibility to curcumin-induced apoptosis has been demonstrated upon IDH2 silencing in HCT116 cells [76]. Cardiac hypertrophy development is attributed to a decrease in IDH2 activity owing to the lipoperoxidation product 4-hydroxynonenal and oxidative stress [77]. IDH2 is also protective for paraquat-mediated oxidative inactivation of aconitase in heart mitochondria [78]. Inactivation of IDH2 activity by various ROS insults [79] is an important factor that has to be accounted for in any consideration of oxidative stress in cells. The forward Krebs cycle activity of IDH2 is inactivated by 4-hydroxynonenal [80], singlet oxygen [81], hypochlorous acid [82], aluminum [83], nitric oxide [84], and peroxynitrite [85]. Peroxynitrite forms S-nitrosothiol adducts on Cys305 and Cys387 of IDH2 under nitrosative stress, such as that established in the liver of ethanol-fed rats [85]. Glycation-mediated IDH2 damage has also been reported [86].

 IDH2 activity first increases and then decreases with age in fibroblasts and liver, kidney, and testes tissues of rats fed *ad libitum* but not of those fed a calorie-restricted diet [87]. Recently, caloric restriction has been proven to act via IDH2 deacetylation through SIRT3 and thus promote an antioxidant role for IDH2-produced NADPH [44]. Again, the activity within the forward Krebs cycle was considered. It is not known whether SIRT3-mediated deacetylation also activates the NADPH-dependent “reverse” reaction, that is, reductive carboxylation.

### 4.2. The Dissipative Isocitrate/2OG Cycle

The dissipative isocitrate/2OG cycle has been suggested based on the reductive carboxylation reaction of IDH2 (counter Krebs cycle reaction direction, NADPH dependent) in conjunction with the forward IDH3 reaction in the canonical Krebs cycle [63]. The cycle may manifests itself in the absence of citrate export from mitochondria, as normally occurs in non-malignant cells, since cycling is impossible when reversed aconitase reaction depletes isocitrate. The cycle would also be possible at sufficient reactant pools and inner membrane energization. Isocitrate formed by the reductive carboxylation reaction of IDH2 is processed back to 2OG by IDH3. Although in non-malignant cells Complex I regenerates NADH to NAD^+^ and NADP^+^ could be regenerated to NADPH by, for example, mitochondrial malic enzyme, with increasing malignancy (more dormant state of mitochondria and hence decreasing respiration), the mitochondrial inner membrane H^+^ transhydrogenase [24, 63, 67] may alternatively transfer electrons from NADH and NADP^+^ to NAD^+^and NADPH at the expense of the proton-motive force [24]. However, it remains to be determined, whether this cycle is possible with d-2-hydroxyglutarate. If d-2-hydroxyglutarate was metabolized by IDH3 in the canonical Krebs cycle, then the cycle would be automatically induced by the appearance of d-2-hydroxyglutarate at simultaneously active H^+^ transhydrogenase. Nevertheless OXPHOS cannot be completely dormant, since proton-motive force would be required for this normal “forward” transhydrogenase reaction [24].

### 4.3. Consequences of Reductive Carboxylation for ROS Homeostasis in Cancer Cells

Consider the situation in highly malignant cells in which energy is derived primarily from glycolysis disconnected from OXPHOS (Warburg phenotype) and high reductive carboxylation glutaminolysis takes place (Figure 2(a)). Presumably, OXPHOS impairment [2] or deep hypoxia [41] may set up this metabolic pattern. In this case, higher glucose-6-phosphate dehydrogenase activity (the first PPP enzyme) produces more NADPH [88]. It may be erroneously considered as antioxidant action; however, because the constitutively expressed NADPH oxidase isoform-4, NOX4 [89, 90], can consume a major portion of the excess NADPH and produce more superoxide and consequently release more H_2_O_2_ into the cytosol, the overall reaction scheme may be prooxidant (Figure 2(a)). This contributes to a much higher oxidative stress state in highly malignant cancer cells. Recently, NOX4 was also suggested to have mitochondrial localization [91]. Probably, NOX4 has K_*m*_ in the same order of magnitude as IDH enzymes. The NOX4 consumption of NADPH leaves fewer redox equivalents for the reduction of cellular glutathione and other redox systems [92–94]. The cytosolic oxidative stress is further intensified by the slow electron transport in low respiring (dormant) mitochondria of highly malignant cancer cells [1–5, 68], resulting in more superoxide release to the cytosol as well as the mitochondrial matrix from the respiratory chain. The ongoing maximum reductive carboxylation reaction further contributes to the oxidative stress by consuming NADPH, thus leaving less NADPH for maintenance of the reduced glutathione pool. Moreover, as mentioned above, the accumulated NADH at slow respiration may lead to NAD^+^ formation in the reversed H^+^ transhydrogenase reaction by concomitant NADPH formation from NADP^+^ to further feed the NADPH pool and hence reductive carboxylation. Simultaneously, NAD^+^ may lead to SIRT3-mediated activation of IDH2, at least of its “forward mode” [44], but it is not known whether reductive carboxylation is also activated by deacetylation of IDH2.

### 4.4. Consequences of IDH2 Reaction within the Forward Krebs Cycle for ROS Homeostasis in Cancer Cells

We next consider an intermediate Warburg phenotype, characterized by the mixed use of sole glycolysis, that is, aerobic glycolysis producing lactate, and OXPHOS (Figure 2(b)). The latter may be represented either by OXPHOS pyruvate metabolism and/or by OXPHOS glutaminolysis. Under these conditions, considerable cytosolic oxidative stress is expected because of the elevated NOX4 activity, as described above. However, a lower mitochondrial contribution to the cytosolic oxidative stress exists owing to an intermediate level of respiration and hence lower superoxide release from mitochondria to both the cytosolic and matrix compartments [92]. There is also lower oxidative stress expected in the matrix owing to the possible ongoing dissipative isocitrate/2OG cycle, which by decreasing the proton-motive force [63] decreases mitochondrial superoxide formation [95]. This can be considered, however, only when citrate efflux from mitochondria is not dominant or when d-2-hydroxyglutarate would be cycling instead of isocitrate/2OG. If this is the case, NAD^+^ rather than NADH would accumulate, further feeding the isocitrate/2OG cycle by simultaneous action of the forward IDH3 reaction and reverse (reductive carboxylation) reaction of IDH2. The accumulated NAD^+^ would also promote SIRT3-mediated IDH2 deacetylation, consequently accelerating the IDH2 branch of the reaction cycle (Figure 2(b)). Conditions similar to those described here may occur at hypoxia.

### 4.5. Regulation of ROS Homeostasis in Nonmalignant Cells: IDH2 Contribution

Finally, we consider the situation in non-malignant cells, in which OXPHOS predominates and the sole glycolysis and PPP activities are low (Figure 2(c)). In this case, low oxidative stress in the cytosol is a consequence of the negligible NOX4 activity and the low contribution of mitochondria to the cytosolic ROS pool. Under these normal conditions, we assume that the forward IDH2 reaction generates NADPH, which further improves the reduced state of the mitochondrial matrix glutathione and peroxiredoxin systems. Indeed, ample evidence suggests that in cells with unattenuated OXPHOS, IDH2 plays an important antioxidant role that is further strengthened by NAD^+^ accumulation in highly respiring cells. NAD^+^ then induces SIRT3-mediated IDH2 deacetylation, thus increasing its protective function in NADPH formation for the maintenance of the reduced glutathione and peroxiredoxin systems and for self-maintenance by the reactivation of cystine-inactivated IDH2 by glutaredoxin-2 [59].

### 4.6. IDH2-Dependent ROS Homeostasis at Glance

(a) Situation in cancer cells with a prevalent Warburg phenotype and high reductive carboxylation. In the cytosol, higher glucose-6-phosphate dehydrogenase activity (G6PDH) produces higher NADPH within the pentose phosphate pathway (PPP). NADPH-oxidase isoform-4 (NOX4) thus produces more superoxide and consequently contributes to high levels of reactive oxygen species (ROS) in the cytosol. The cytosolic oxidative stress is further intensified by the slow electron transport in low-respiring (dormant) mitochondria, leading to higher superoxide release to the cytosol and matrix compartments. The ongoing maximum reductive carboxylation reaction further contributes to oxidative stress by consuming NADPH, thus leaving less for maintenance of the reduced glutathione pool. The accumulated NADH at slow respiration may lead to NAD^+^ formation in the reverse H^+^ transhydrogenase **(**TH**)** reaction (due to low proton/motive force) with concomitant NADPH formation from NADP^+^ to further feed the NADPH pool and hence reductive carboxylation. Hypothetically, NAD^+^ may lead to sirtuin 3 (SIRT3)-mediated deacetylation/activation (+) of IDH2, but it is not known whether reductive carboxylation is also activated by deacetylation of IDH2. (b) Situation in cancer cells with an intermediate Warburg phenotype and possible OXPHOS glutaminolysis. The major contribution to the cytosolic oxidative stress is the same as described above. However, a lower mitochondrial contribution to cytosolic ROS leads to intermediate oxidative stress under these conditions. Indeed, an intermediate or high respiration leads to much lower superoxide production and release from the mitochondria to both the cytosolic and matrix compartments (dashed arrows). Lower oxidative stress is also expected in the matrix owing to the ongoing dissipative isocitrate/2OG cycle, which decreases further mitochondrial superoxide formation by decreasing the proton-motive force. OXPHOS glutaminolysis may predominate under these conditions; hence, RCG might not be completed and the isocitrate/2OG cycle with forward H^+^ transhydrogenase reaction may be initiated. NAD^+^ would accumulate owing to high respiration, further feeding the isocitrate/2OG cycle. The accumulated NAD^+^ would also promote SIRT3-mediated deacetylation/activation of IDH2. (c) Situation in nonmalignant cells. Aerobic glycolysis and PPP activity are low; consequently, low oxidative stress in the cytosol also results from the negligible NOX4 activity and the low contribution of mitochondria to the cytosolic ROS. The forward IDH2 reaction thus forms NADPH, which further improves the reduced state in the mitochondrial matrix glutathione and peroxiredoxin systems. As in (b), the NAD^+^ accumulation in highly respiring cells then induces SIRT3-mediated deacetylation/activation of IDH2.

## 5. Hypothetical IDH2 Involvement in Redox Signaling

As briefly described above, mutant IDH2 as well as IDH1 [96–100] produce d-2-hydroxy-glutarate, which can initiate an HIF-mediated “hypoxic type” of gene reprogramming even at normoxia [58]. Nevertheless, detail investigations of d-2-hydroxyglutarate effects on HIF signaling are required, since recently an opposite effect, diminishing HIF levels by inhibition of EGLN prolyl 4-hydroxylases, has been reported [99]. Also, non-mutated IDH2 acting in the 2OG/isocitrate cycle together with H^+^ transhydrogenase [63] could contribute to the modulation of the ROS pool by initiating an impulse originating from Complex III to dissipate the proton-motive force, which reduces superoxide formation in the mitochondrial respiratory chain [95]. In contrast to mutant IDH2 activity, the ongoing dissipative 2OG/isocitrate cycle in the absence of citrate efflux from mitochondria would retard HIF signaling.

## 6. Future Perspectives

The discovery of mutant IDH2 and IDH1 in certain gliomas and AML and their production of the oncogenic metabolite d-2-hydroxyglutarate have unraveled a fascinating story of cancer self-acceleration via intermittent episodes of genome instability and metabolic remodeling and namely via epigenomic alterations [98, 100]. However, there are additional aspects to be clarified. First, the exact role of d-2-hydroxy-glutarate must be further investigated to determine whether it promotes the reverse carboxylation mode of glutaminolysis and whether it acts in the dissipative 2OG/isocitrate cycle, which would then become the 2OG/d-2-hydroxyglutarate cycle, as we can now only speculate. Also, conditions under which d-2-hydroxyglutarate might be formed in non-mutant IDH2 should be defined. Second, the role of SIRT3 has to be established to determine whether it prevents or accelerates malignancy via IDH2. In particular, the question whether SIRT3 activates reductive carboxylation must be resolved. Finally, the role of IDH2 in other cancer types distinct from AML, gliomas, and renal tumors of hereditary leiomyomatosis should be investigated.

## Figures and Tables

**Table 1:** Kinetics of IDH isoforms as compared to homodimeric mutant enzymes with clinically relevant mutations. Unless specified “reductive”, forward reactions were measured. “n.d.”: not determined.

Enzyme	Organism/tissue	Mutation	conditions	K_*m*_ isocitrate (*μ*M)	K_*l*_, 2-oxo-glutarate (*μ*M)	K_*m*_ NADP^+^ (*μ*M)	*V*_max⁡_ (*μ*mol·min^−1^·mg^−1^)	Reference
IDH1	Human	wt		65	1900	49	44000 s^−1^	[96]
	Human	R132H		370	24	84	38 s^−1^	[96]
	Human	R132H	Reductive	2OG: 965	n.d	NADPH: 0.44	1000 s^−1^	[96]
	Human	wt		6.4	n.d.	n.d.	14	[101]
	Human	R132H		1280	n.d.	n.d.	0.8	[101]
	Rat liver	wt		120	n.d.	150	70	[102]

IDH2	Porcine	wt		8.4	n.d.	5.6	43	[103]
	Porcine	wt		6	n.d.	5	40	[51]
	Porcine	R133Q		990	n.d.	11	21	[51]
	Rat liver	wt		70	n.d.	60	66	[102]
	Rat heart	wt		45	80	46	16	[104]
	Rat heart	wt	Ischemic	17	250	46	38	[104]

IDH3	Human	wt	No ADP	2000	n.d.	NAD^+^: 60	26	[105]
	Human	wt	+1 mM ADP	50	n.d.	n.d.	n.d.	[105]
